# Dietary quercetin potentiates the antiproliferative effect of interferon-α in hepatocellular carcinoma cells through activation of JAK/STAT pathway signaling by inhibition of SHP2 phosphatase

**DOI:** 10.18632/oncotarget.22556

**Published:** 2017-11-20

**Authors:** Ighodaro Igbe, Xiao-Fei Shen, Wei Jiao, Zhe Qiang, Teng Deng, Sheng Li, Wan-Li Liu, Han-Wei Liu, Guo-Lin Zhang, Fei Wang

**Affiliations:** ^1^ Key Laboratory of Natural Medicine and Clinical Translation, Chengdu Institute of Biology, Chinese Academy of Sciences, Chengdu, China; ^2^ Department of Pharmacology and Toxicology, Faculty of Pharmacy, University of Benin, Benin City, Nigeria; ^3^ MOE Key Laboratory of Protein Sciences, Collaborative Innovation Center for Diagnosis and Treatment of Infectious Diseases, School of Life Sciences, Tsinghua University, Beijing, China; ^4^ Ningbo Entry-Exit Inspection and Quarantine Bureau Technical Center, Ningbo, China

**Keywords:** quercetin, interferon, SHP2, JAK, STAT

## Abstract

Type I interferons (IFN-α/β) have broad and potent immunoregulatory and antiproliferative activities, which are negatively regulated by Src homology domain 2 containing tyrosine phosphatase-2 (SHP-2). Inhibition of SHP2 by small molecules may be a new strategy to enhance the effcacy of type I IFNs. Using an *in vitro* screening assay for new inhibitors of SHP2 phosphatase, we found that quercetin was a potent inhibitor of SHP2. Computational modeling showed that quercetin exhibited an orientation favorable to nucleophilic attack in the phosphatase domain of SHP2. Quercetin enhanced the phosphorylation of signal transducer and activator of transcription proteins 1 (STAT1) and promoted endogenous IFN-α-regulated gene expression. Furthermore, quercetin also sensitized the antiproliferative effect of IFN-α on hepatocellular carcinoma HepG2 and Huh7 cells. The overexpression of SHP2 attenuated the effect of quercetin on IFN-α-stimulated STAT1 phosphorylation and antiproliferative effect, whereas the inhibition of SHP2 promoted the effect of quercetin on IFN-α-induced STAT1 phosphorylation and antiproliferative effect. The results suggested that quercetin potentiated the inhibitory effect of IFN-α on cancer cell proliferation through activation of JAK/STAT pathway signaling by inhibiting SHP2. Quercetin warrants further investigation as a novel therapeutic method to enhance the efficacy of IFN-α/β.

## INTRODUCTION

Type 1 interferons including IFN-α and IFN-β are a family of cytokines which induce and activate the Janus kinase/signal transducer and activator of transcription (JAK/STAT) pathway resulting in their antiviral, antiproliferative, and antitumor activities as well as regulate the activity of the immune system [1]. Several negative regulators of JAKs and STATs participate in the attenuation of JAK/STAT pathway signaling, including Src homology domain 2 containing tyrosine phosphatase-1/2 (SHP-1/2), suppressor of cytokine signaling (SOCS) family members, protein inhibitor of activated STAT family members, and the ubiquitin/26S proteasome pathway [2]. Type I IFNs have been used clinically to treat a variety of cancers and viral diseases; however, several serious side effects such as hepatic and hematologic toxicities are related to IFNs therapy. The dose and duration of IFNs therapy also appears to be related to the severity of these side effects [3]. Recently, cell-based ISRE luciferase reporter screening identified several small molecules that were able to modulate the JAK/STAT signaling via various mechanisms, leading to their inhibition of proliferation and viral replication [4–6]. Therefore, the identification of novel bioactive molecues that can enhance or imitate the actions of type I IFNs by inhibition of the negative regulators of JAK/STAT pathway will lead to new methods being developed for treatment of various diseases using IFNs, thus allowing the administration of decreased IFN dosage and the subsequent alleviation of its side effects.

SHP2, encoded by *PTPN11*, is a first identified oncogenic tyrosine phosphatase [7] that been linked to several genetic, developmental diseases such as Noonan syndrome and multiple cancers including leukemia, lung, and breast cancer, and neuroblastoma [8]. SHP2 is expressed in most tissues and play a regulatory role in cell survival and proliferation by activating the RAS-ERK signaling pathway [9], and is involved in the programmed cell death 1 (PD-1) and B- and T-lymphocyte attenuator (BTLA) immune checkpoint pathways [10]. Furthermore, SHP2 also plays a key role in the negative regulation of the IFN-induced JAK/STAT pathway [11]. In SHP2 knockout mouse fibroblasts, treatment with IFN-γ and IFN-α resulted in elevated tyrosine phosphorylation of STAT1 and STAT2 and augmented the suppression of cell viability [12]. SHP2 regulates STAT1 activity mainly by blocking of JAK tyrosine kinase activity and the dephosphorylation of STAT1 at both the tyrosine and serine residues [13]. The inhibition of SHP2 activity suppressed tumor growth and has been an emerging target of cancer treatment [7–9]. Small molecules have been found to inhibit SHP2 activity; however, their efficacy in the enhancement of type I IFN signaling is still unclear.

The abundance of flavonoids in vegetables and fruits contributes to their importance in the reduction of the risk of many chronic diseases [14]. However, their influence on the JAK/STAT pathway, the dominant anticancer and antiviral system in human body, is rarely studied. Quercetin (3, 3′, 4′, 5, 7-pentahydroxylflavone) is the most abundant flavonoid in the human diet and it is present at high concentrations in onions, apples, red wine, and a variety of berries [15]. Previous studies have shown that quercetin specifically exerted antineoplastic activity through an influence on proliferation, differentiation, and apoptosis in a variety of tumor cell types, including liver cancer [16, 17]. Mechanismly, quercetin modulated several signal transduction pathways involving MEK/ERK, NF-κB, Nrf2/keap1, PI3K/Akt/mTOR, and Wnt/β-catenin, which are associated with the processes of inflammation and carcinogenesis [18]. However, the pleiotropic actions of quercetin warrants further evaluation of other proteins that may be in the mechanisms of action of quercetin.

Previously, we identified that quercetin increased expression of antiviral genes namely 2′ 5′oligoadenylate synthetase (2′5′-OAS) and RNA-activated protein kinase (PKR) regulated by type I IFNs by activation of the JAK/STAT pathway [5]. However, the mechanism of action on the JAK/STAT pathway remains elusive. In the present study, we investigated whether the activation of quercetin on type I IFN-induced JAK/STAT signaling was due to inhibition of SHP2.

## RESULTS

### Inhibitory effect of quercetin on SHP2 activity and expression

**Figure 1:**
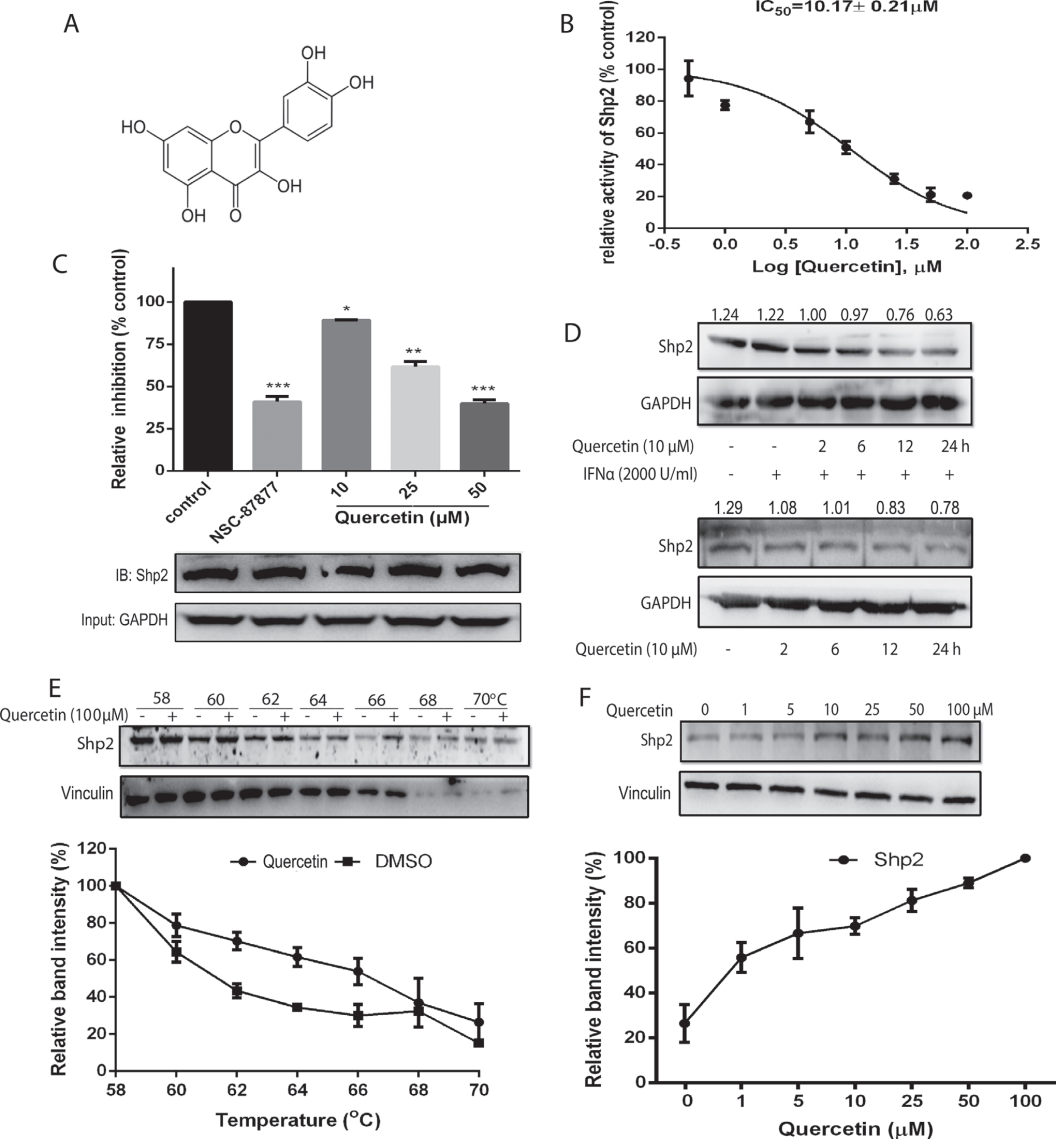
Inhibitory effect and interaction of quercetin with SHP2 activity **(A)** The chemical structure of quercetin. **(B)** DiFMUP substrate was treated with various concentrations of quercetin in the presence of recombinantly expressed SHP2 protein and the IC_50_ was calculated. **(C)** HEK293A cells treated with quercetin (10, 25, 50 μΜ) and SHP2 inhibitor (NSC-87877, 10 μM) for 6 h were lysed, immunoprecipitated with anti-SHP2 antibody, and SHP2 activity was determined. Then, the immunoprecipitation products were used to quantify the protein amount of SHP2. GAPDH was used as the input. **(D)** HepG2 cells were treated with quercetin (10 μM) alone for 2, 6, 12, and 24 h, or followed by the addition of 2000 U/mL IFN-α for 30 min. The cell lysates were immunoblotted with the anti-SHP2 antibody. GAPDH staining is shown as a loading control. **(E, F)** The cellular thermal shift assay was performed on HEK293A cells as described in Materials and Methods section. The stabilization effect of quercetin on SHP2 and vinculin at different temperatures (E) and different concentrations (F) was evaluated by western blot. Each experiment was repeated at least three times. Data were expressed as mean ± S.D., ^*^p < 0.05, ^**^p < 0.01, ^***^p < 0.001 *vs* control.

By using the recombinantly expressed SHP2 protein, a screening assay was used to screen a natural products based library containing 1431 compounds [5]. After hit reconfirmation, quercetin was shown to significantly inhibit SHP2 activity. The chemical structure of quercetin is shown in Figure 1A. Quercetin showed a concentration-dependent inhibition on the activity of SHP2 with an IC_50_ value of 10.17 ± 0.21 μM *in vitro* (Figure 1B). To determine the effect of quercetin on SHP2 activity, HEK293A cells were incubated with quercetin. The cell lysates were then immunoprecipitated with the anti-SHP2 antibody and the activity was determined. Quercetin significantly inhibited SHP2 activity in the treated cells in a concentration-dependent manner compared with the untreated cells (Figure 1C). In addition, quercetin also significantly decreased the protein expression of SHP2 in HepG2 cells in the presence or absence of IFN-α (Figure 1D). In order to know whether quercetin interacted with SHP2 in cells, we performed a cellular thermal shift assay, which is a newly developed method to evaluate drug binding to target proteins in cells [19]. Compared with the DMSO control, the presence of quercetin markedly increased the accumulation of SHP2 in the soluble fraction at the temperatures examined, as shown in Figure 1E. We also tested the concentration-response of quercetin on SHP2 stability at increased temperatures. An increase in quercetin concentration resulted in the markedly increased accumulation of SHP2, as shown in Figure 1F. These data suggested that quercetin interacted directly with SHP2 and inhibited the activity of SHP2 in cells. We further evaluated whether quercetin affects the activity and stability of SHP1. Quercetin inhibited the activity of SHP1 in a concentration-dependent manner with a calculated IC_50_ value of 31.14 ± 1.27 μM *in vitro* (Supplementary Figure 1A), and increased the thermal stability of SHP1 (Supplementary Figure 1B). These results suggest that quercetin interacted directly with both SHP2 and SHP1 and inhibited the activity of SHP2 more potently than SHP1.

### Molecular docking of quercetin with SHP2

**Figure 2:**
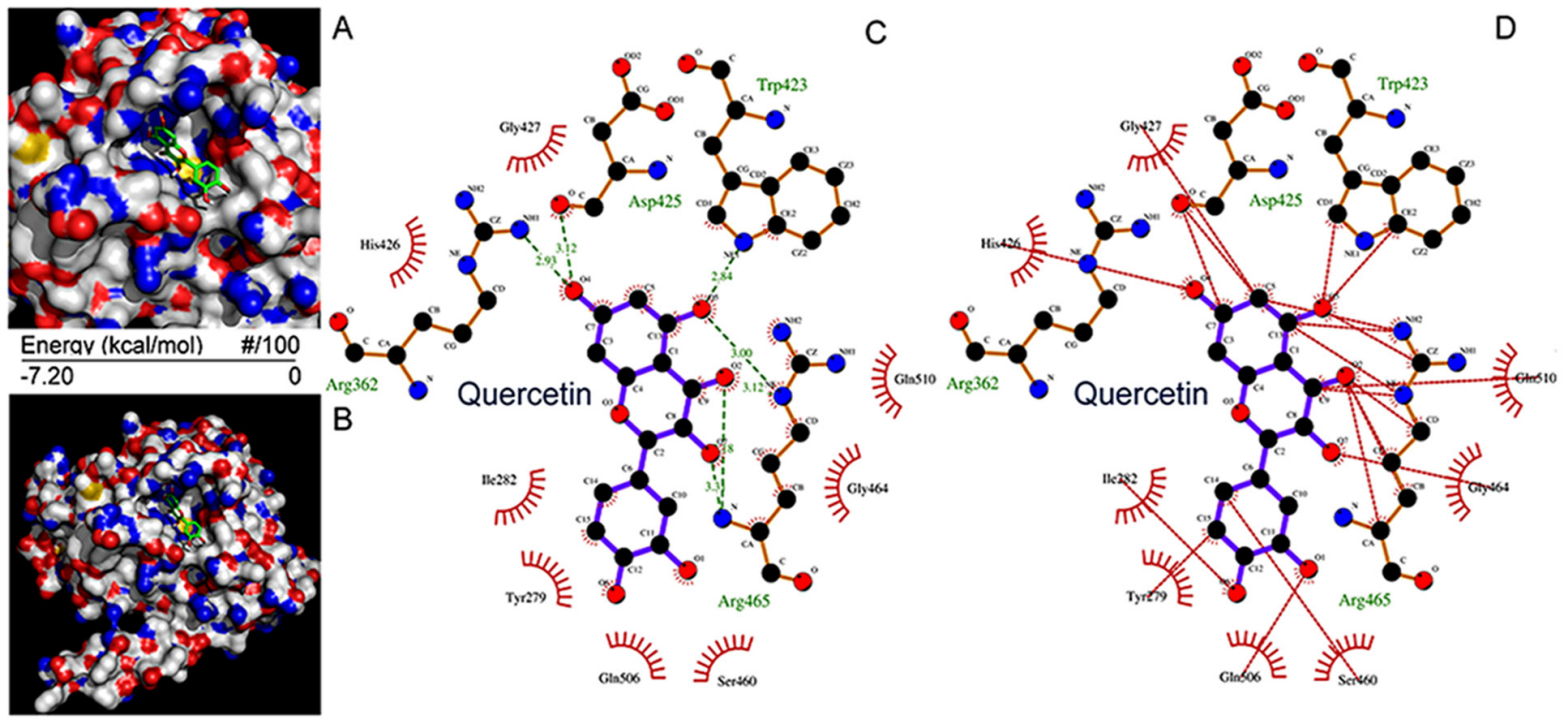
Molecular docking of quercetin on SHP2 **(A, B)** Quercetin was docked into the SHP2 active site (PTP domain) using Autodock 4.2.6. Quercetin is shown using stick model and the carbon atoms are colored in green. **(C, D)** The docking scheme of quercetin and SHP2 PTP domain. The hydrogen bonds formed between quercetin and the protein via Arg362, Trp423, Asp425, and Arg465 are highlighted in green dotted lines, while the hydrophobic interactions formed between quercetin and the SHP2 PTP domain via Tyr279, Ile282, Trp423, Asp425, His426, Gly427, Ser460, Gly464, Arg465, Gln506, and Gln510 are highlighted in red dotted lines.

The quercetin binding sites in SHP2 were assessed using computer docking analysis and it was determined that quercetin fitted well in the active site of SHP2 (Figure 2A and 2B). To determine the binding modes of quercetin to the active site of SHP2, the hydrogen-bond (H-bond) formation and hydrophobic interactions between quercetin and the PTP domain of SHP2 were evaluated. There are three polar hydrogens and one carbonyl-oxygen in quercetin that are involved in H-bonding with the Arg^362^, Trp^423^, Asp^425^, Arg^465^ residues of SHP2 (Figure 2C). Furthermore, quercetin also formed hydrophobic interactions with the residues Tyr^279^, Ile^282^, Trp^423^, Asp^425^, His^426^, Gly^427^, Ser^460^, Gly^464^, Arg^465^, Gln^506^, and Gln^510^ in the PTP domain (Figure 2D), which may also contribute to its inhibition of the phosphatase activity of SHP2. These results suggested that the orientation/conformation exhibited by quercetin in the PTP domain of SHP2 allowed it to be subjected to nucleophilic attack.

### Quercetin enhances type I IFNs-induced activation of JAK/STAT pathway

**Figure 3:**
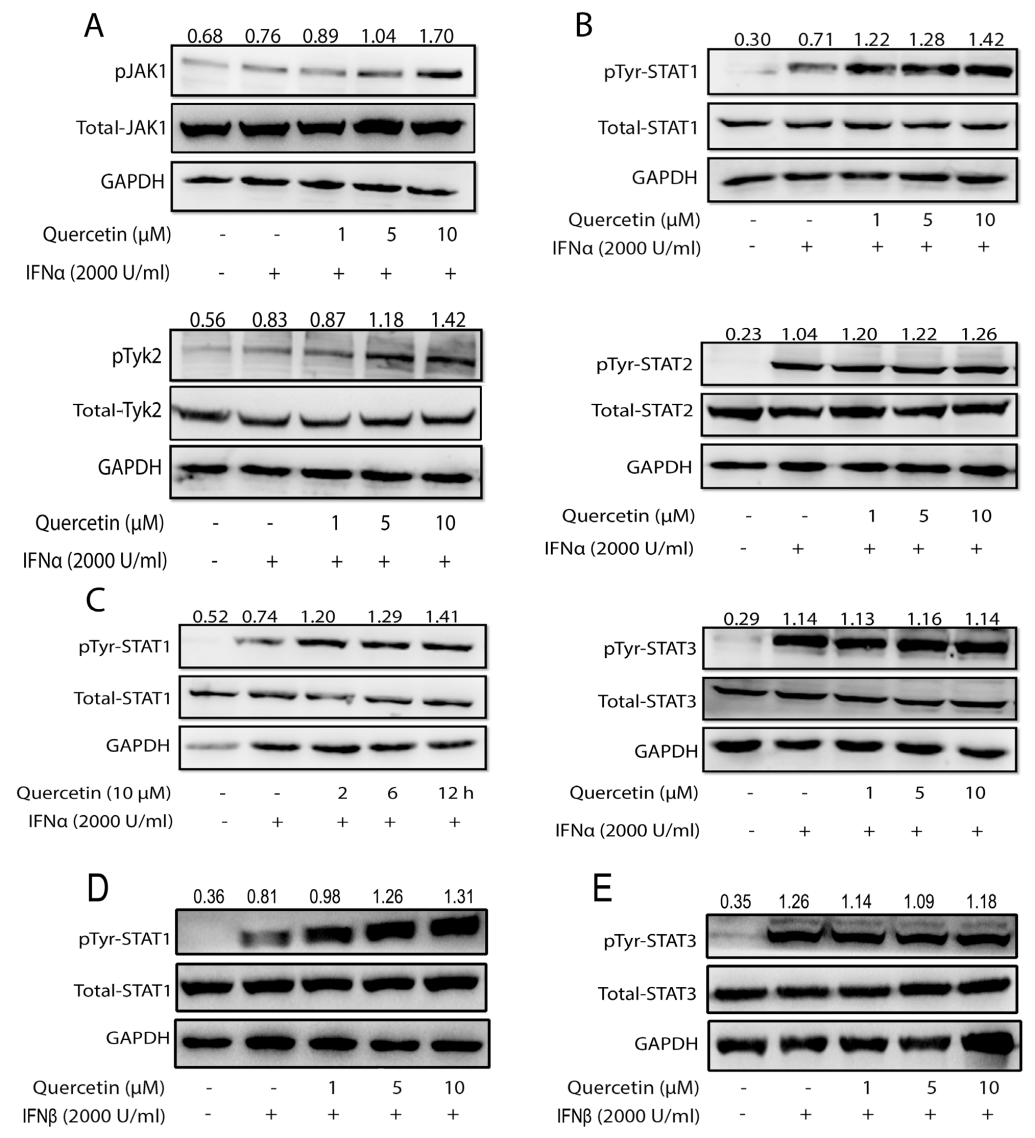
Quercetin enhances IFNα-induced JAK/STAT pathway activation **(A, B)** HepG2 cells grown in six-well plates were treated with quercetin (1, 5, or 10 μM) for 6 h, followed by the addition of 2000 U/mL IFN-α for 30 min. The cell lysates were immunoblotted with phospho-JAK1 (Tyr1022/1023), phospho-Tyk2 (Tyr1054/1055) antibodies, phospho-STAT1 (Tyr701), phospho-STAT2 (Tyr690), phospho-STAT3 (Tyr705) antibodies, JAK1, Tyk2, STAT1, STAT2, and STAT3 antibodies. GAPDH staining is shown as a loading control. **(C)** HepG2 cells were treated with quercetin (10 μM) for 2, 6, and 12 h, followed by the addition of 2000 U/mL IFN-α for 30 min. The cell lysates were immunoblotted with phospho-STAT1 (Tyr701) or STAT1. GAPDH staining is shown as a loading control. **(D, E)** HepG2 cells were treated with quercetin (10 μM) for 2, 6, and 12 h, followed by the addition of 2000 U/mL IFN-β for 30 min. The cell lysates were immunoblotted with phospho-STAT1 (Tyr701) and phospho-STAT3 (Tyr705). GAPDH staining is shown as a loading control.

Due to the immense role of SHP2 in the negatively modulating the JAK/STAT pathway signaling, we hypothesized that the inhibition of SHP2 by quercetin will lead to enhanced activation of the JAK/STAT pathway. To test this hypothesis, we initially evaluated the effect of quercetin on IFN-α-induced JAK/STAT signaling. Treatment with only quercetin produced no obvious effect on the tyrosine phosphorylation of STAT1 in HepG2 cells, but significantly promoted tyrosine phosphorylation of STAT1 in the presence of IFN-α (Supplementary Figure 2), which indicated that the activation of the JAK/STAT signaling may be required for quercetin to exert its promotive effect. We then examined the effect of quercetin on the activation of JAKs and STATs in the presence of IFN-α. Compared with only IFN-α, quercetin enhanced the tyrosine phosphorylation of Jak1 and Tyk2 induced by IFN-α (Figure 3A). Thereafter, the effect of quercetin on the phosphorylation of STAT1, STAT2, and STAT3 in the presence of IFN-α was evaluated. Compared to IFN-α alone, quercetin significantly enhanced the tyrosine phosphorylation of STAT1, but had no significant effect on the tyrosine phosphorylation of STAT2 and STAT3 (Figure 3B). Quercetin also promoted the tyrosine phosphorylation of STAT1 in a time-dependent manner (Figure 3C). Moreover, quercetin obviously elevated IFN-β-induced tyrosine phosphorylation of STAT1 (Figure 3D), but not STAT3 (Figure 3E). These results suggested that quercetin enhanced the activation of type I IFNs-induced JAK/STAT pathway. Interestingly, tyrosine phosphorylation of STAT1 (Supplementary Figure 3A) but not STAT3 (Supplementary Figure 3B) could be remarkably enhanced by quercetin treatment in the presence of IFN-γ, indicating that quercetin also boosted the activation of type II IFN-induced signaling.

### Quercetin promotes downstream genes expression induced by IFN-α

**Figure 4:**
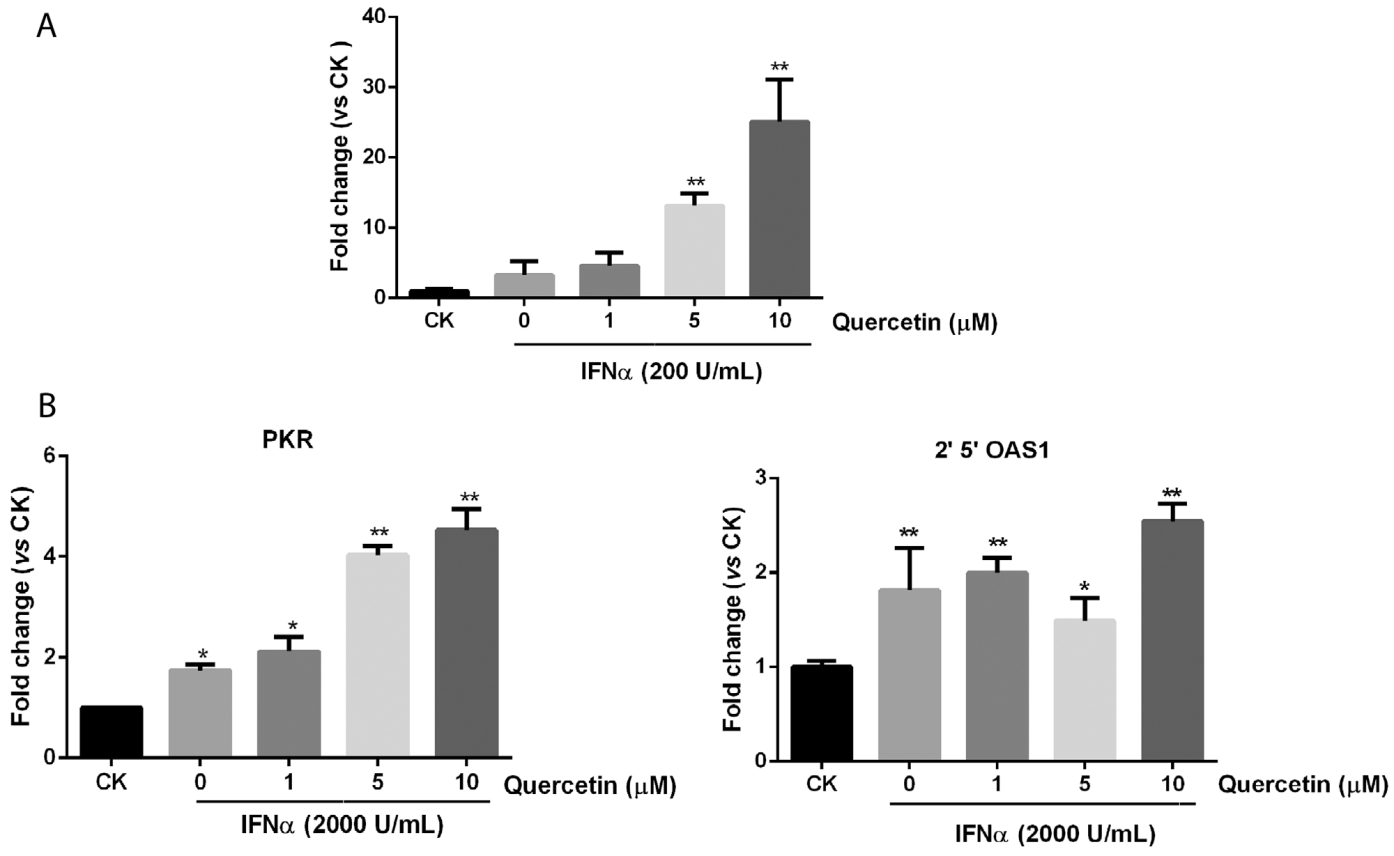
Quercetin promotes downstream genes expression in JAK/STAT pathway **(A)** HepG2-ISRE-Luc2 cells were seeded in 96-well plates (1×10^4^ cells/well) and pretreated with various concentrations of quercetin for 2 h before 200 U/mL IFN-α was added for a further 24 h. The luciferase activity of the total cell lysate was measured. **(B)** HepG2 cells were treated with various concentrations of quercetin for 6 h before the addition of 2000 U/mL IFN-α for 30 min. Real-time quantitative polymerase chain reaction was used to determine the messenger RNA expression of PKR or 2′,5′-OAS. The result is presented as induction (n-fold) relative to basal levels in untreated cells. GADPH was used as an internal control. ^*^p < 0.05, ^**^p < 0.01 vs control; CK, DMSO control.

To examine whether quercetin can increase downstream gene expression induced by IFN-α, we first assessed the effect of quercetin on ISRE reporter expression. In Figure 4A, quercetin significantly promoted ISRE reporter expression in the presence of IFN-α, compared with IFN-α alone, in a dose-dependent manner. Furthermore, IFN-α alone or IFN-α plus quercetin treatment for 24 h, did not affect the cell viability of HepG2-ISRE-Luc2 cells (Supplementary Figure 4). The mRNA expression of two IFN-α responsive genes, 2′,5′-OAS and PKR, were determined after treatment with various concentrations of quercetin. The mRNA levels of 2′,5′-OAS and PKR were significantly increased after treatment with quercetin plus IFN-α compared with only IFN-α, as shown in Figure 4B. These results supported our previous results that quercetin enhanced the downstream endogenous gene expression induced by IFN-α.

### Quercetin enhances the antiproliferative effect of IFN-α on cancer cells

**Figure 5:**
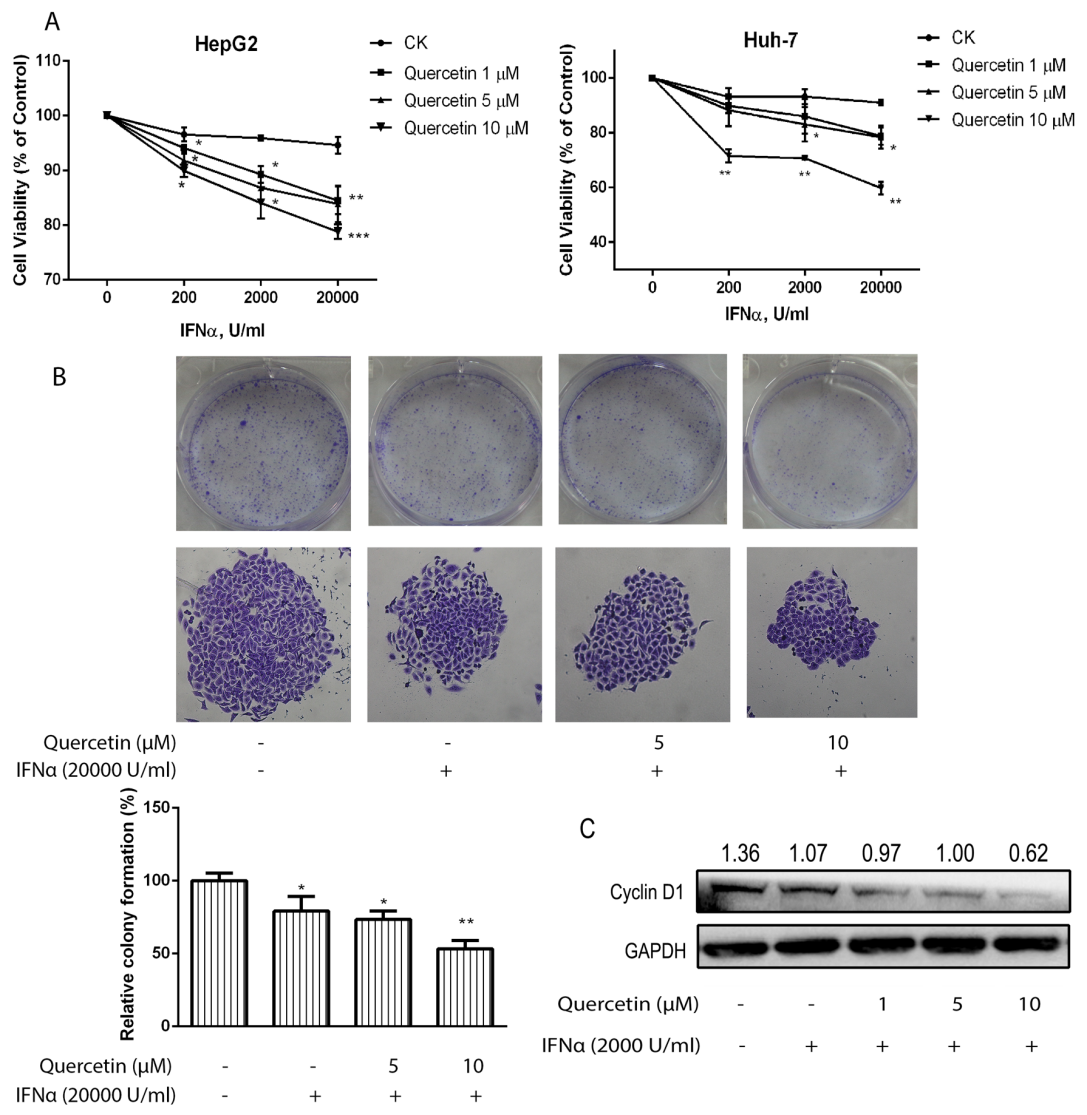
Quercetin enhances the inhibitory effect induced by IFN-α on cancer cell viability **(A)** HepG2 and Huh-7 cells were seeded in 96-well plates at 0.5 × 10^4^ cells/well and treated with various concentrations of IFN-α and quercetin for 72 h. Cell viability was measured using the Alamar Blue assay and the values are expressed as the percentage cell viability relative to the DMSO control. **(B)** HepG2 cells grown in six-well plates were treated with quercetin (5 and 10 μM) and IFN-α (2 × 10^4^ U/mL) for 10 days and colonies were visualized by staining with crystal violet and counted manually. Data are expressed as a percentage of the colonies in the control cells and are the mean ± S.D. of two experiments, both of which were performed in duplicate. ^*^p < 0.05, ^**^p < 0.01, ^***^p < 0.001 vs control; CK, DMSO control. **(C)** HepG2 cells were treated with IFN-α and quercetin for 72 h. The cell lysates were immunoblotted with cyclin D1 and GAPDH antibodies.

To determine whether quercetin could sensitize cancer cells to IFN-α-induced antiproliferative effect, we treated human hepatoma cancer cells (HepG2 and Huh-7) with quercetin and IFN-α. The addition of quercetin (1–10 μM) potentiated the antiproliferative effect of IFN-α in a dose-dependent manner (Figure 5A); however, quercetin alone did not affect the cell proliferation at the same concentration (Supplementary Figure 5). Furthermore, quercetin significantly inhibited the colony formation of HepG2 cells in the presence of IFN-α when compared to cells treated with only IFN-α (Figure 5B). In addition, the suppression of IFN-α on cyclin D1 expression was further enhanced by quercetin treatment (Figure 5C). These results suggested that quercetin enhanced IFN-α-induced antiproliferative effect in hepatocellular carcinoma cells.

### Quercetin promotes IFN-α-mediated effects via SHP2 inhibition

**Figure 6:**
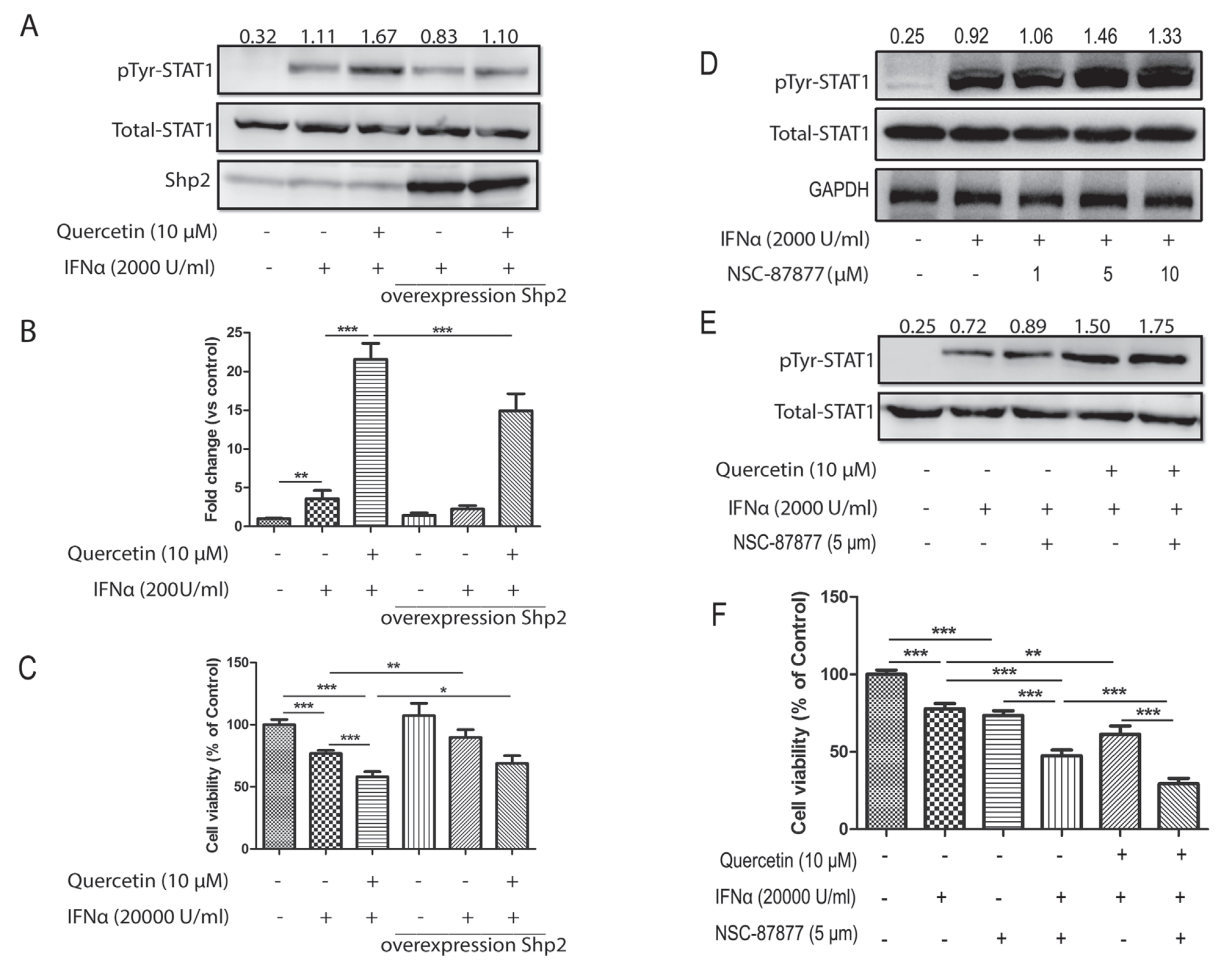
Quercetin increases IFN-α-induced phosphorylation of STAT1 via SHP2 inhibition **(A)** HepG2 cells were transiently transfected with the empty plasmid or pCMV-SHP-2 plasmid using Trans-EZ transfection reagent, respectively. After 24 h, the cells were treated with quercetin (10 μM) for 6 h before the addition of 2000 U/mL IFN-α for 30 min. The cell lysates were immunoblotted with phospho-STAT1 (Tyr701), STAT1, and SHP2 antibodies. **(B)** HepG2-ISRE-Luc2 cells were transfected with empty or SHP2 expression vectors, and then seeded in 96-well plates (1×10^4^ cells/well) and pretreated with quercetin (10 μM) for 2 h before 200 U/mL IFN-α was added for a further 24 h. The luciferase activity of the total cell lysate was measured. (**C**) HepG2 cells were transiently transfected with the pCMV-SHP-2 plasmid. After 24 h, cells were seeded in 96-well plates at 0.5 × 10^4^ cells/well and treated with 20000 U/mL IFN-α and quercetin (10 μM) for 72 h. Cell viability was measured using the Alamar Blue assay and the values are expressed as the percentage cell viability relative to the DMSO control (mean ± S.D). **(D)** HepG2 cells were pretreated with SHP2 inhibitor (NSC-87877, 1, 5, and 10 μM) for 6 h before the addition of 2000 U/mL IFN-α for 30 min. The cell lysates were immunoblotted with phospho-STAT1 (Tyr701) and STAT1 antibodies. **(E)** HepG2 were seeded in six-well plates were pretreated with quercetin (10 μM) or SHP2 inhibitor (NSC-87877, 5 μM) for 6 h before the addition of 2000 U/mL IFN-α for 30 min. The cell lysates were immunoblotted with phospho-STAT1 (Tyr701) and STAT1 antibodies. **(F)** HepG2 cells were seeded in 96-well plates at 0.5 × 10^4^ cells/well and treated with 20000 U/mL IFN-α, quercetin (10 μM), and NSC-87877 (5 μM) for 72 h. Cell viability was measured using the Alamar Blue assay and the values are expressed as the percentage cell viability relative to the DMSO control (mean ± S.D.). ^*^p < 0.05, ^**^p < 0.01, ^***^p < 0.001 *vs* control.

SHP2 negatively regulates the JAK/STAT pathway signaling initiated by type I IFNs through the dephosphorylation of activated JAKs and STATs. Hence, quercetin may target SHP2 to promote the activation of the JAK/STAT pathway. To test this hypothesis, we overexpressed SHP2 in HepG2 cells, which attenuated the quercetin-induced enhancement of INF-α-induced STAT1 phosphorylation in untransfected cells, as shown in Figure 6A. Meanwhile, overexpression of SHP2 partially abolished IFN-α and IFN-α plus quercetin-induced ISRE reporter expression in HepG2-ISRE-Luc cells (Figure 6B) and viability of HepG2 cells (Figure 6C). To further confirm the role of SHP2 on quercetin-enhanced STAT1 phosphorylation and antiproliferative effect, HepG2 cells were pre-treated with a specific SHP2 inhibitor (NSC-87877). NSC-87877 treatment increased the IFN-α-stimulated phosphorylation of STAT1 (Figure 6D), which was further promoted by addition of quercetin (Figure 6E). NSC-87877 treatment had no significant effect on the tyrosine phosphorylation of STAT2 and STAT3 (Supplementary Figure 6). NSC-87877 treatment also increased IFN-α-induced anti-proliferative effect on HepG2 cells, which was further promoted by addition of quercetin (Figure 6F). As SOCS1 and SOCS3 are also involved in the negative regulation of JAK activation, the effect of quercetin on SOCS1 and SOCS3 expression was evaluated; the results showed that quercetin had no effect on the expression of SOCS1 and SOCS3 (Supplementary Figure 7). We then examined the effect of quercetin on 26S proteasome, another negative regulator of JAK/STAT pathway. As shown in Supplementary Figure 8, quercetin had no inhibitory effect on 26S proteasome. These results suggested that quercetin promotes IFN-α-induced phosphorylation of STAT1 and antiproliferative effect via SHP2 inhibition.

## DISCUSSION

The upregulation of SHP2 expression has been reported in many human cancers; a decrease in SHP2 activity inhibits tumor cell growth and is a promising target for chemotherapy [8–10]. In our study, we found that quercetin, a naturally occurring flavonoid, was a potent inhibitor of SHP2 catalytic activity and suppressed the expression of SHP2 in hepatocellular carcinoma cells. These results were also supported by the finding that quercetin decreased the expression of SHP2 and subsequently downregulated the activation of epidermal growth factor receptor (EGFR) in HeLa cervical cancer cells [20]. The molecular docking of quercetin to the PTP domain responsible for the phosphatase activity of SHP2 showed that quercetin might suppress the phosphatase activity mainly via hydrogen bonding and hydrophobic interaction. Among the amino acid residues that interacted with quercetin, Arg^362^ was considered to enhance the interaction of SHP2 with its substrate proteins and thereby aid opening [21]. Asp^425^ acts as a proton donor for the phenolate leaving group of the substrate and an acceptor during the hydrolysis of the cysteinyl phosphate intermediate [22]. Arg^465^, a key residue of the PTP signature motif (C^459^(X)_5_R^465^), has been associated with the catalysis of SHP2 to stabilize the phosphotyrosine substrate-enzyme complex [23]. NSC-87877, a known SHP2 inhibitor, forms hydrogen bonds with the backbone NH group of Arg^465^ in the PTP signature motif and with the side chains of Lys^280^ and Asn^281^ [24], which are similar to the amino acids predicted for quercetin. Therefore, it is likely that quercetin inhibited the phosphatase activity of SHP2 by forming hydrogen bonds with the key amino acid residues of the PTP domain, including Arg^362^, Trp^423^, Asp^425^, and Arg^465^. Furthermore, some mutations of SHP2, including Tyr279Cys, Ile282Val, and Gln510Glu, have been reported to be involved in the incidence of Noonan syndrome [25]. Quercetin is predicted to be able to form hydrophobic interactions with these amino acids, which suggested that quercetin may inhibit the phosphatase activity of SHP2 by forming hydrophobic interactions with these key amino acid residues of PTP domain. Many dietary flavonoids share a similar structure with quercetin; therefore, it will be of interest to perform further investigations into the structure-activity relationship of flavonoids for the inhibition of SHP2, which may allow the development of new SHP2 inhibitors.

The majority of the current antiviral drugs usually exert their effect by interacting with viral proteins, however, they become less effective with usage due to genetic changes in the viruses [26]. Since, the JAK/STAT pathway plays an important role in antiviral defense and proliferation in human cells, it becomes a promising target for research into novel antiviral and antitumor drugs. Although there is abundance of natural compounds that inhibit the JAK/STAT signaling pathway, information on compounds that activate this pathway is still insufficient [27]. Our results showed that quercetin enhanced IFN-α-induced JAK/STAT pathway and thereby boosting the antiproliferative effect of IFN-α. These results were consistent with our previous report, which showed that quercetin activated the JAK/STAT pathway and increased mRNA expression of two IFN-α responsive antiviral genes, 2′,5′-OAS and PKR [5]. Quercetin activated Jak1 and Tyk2, which led to the observed STAT1 activation, but had no significant effect on the tyrosine phosphorylation of STAT2 and STAT3. STAT1 has been reported as a potential tumor suppressor and regulator of the anti-proliferative/pro-apoptotic responses in tumor cells [28]. The quercetin-induced activation of STAT1 inhibited cell proliferation, which may lead to the observed potentiation of the IFN-α-induced antiproliferative effect in HepG2 and Huh7 cells. However, quercetin had no effect on SOCS1/3 and 26S proteasome, the key negative regulators of JAK/STAT [29]. Thus, quercetin-activated JAK/STAT signaling is probably not mediated by SOCS1/3 and 26S proteasome; instead, it might inactivate a protein phosphatase that dephosphorylates JAKs. In the present study, quercetin was identified as an inhibitor of protein phosphatase SHP2 that causes suppression of the dephosphorylation of Jak1 and Tyk2, which in turn causes the sustained activation of STAT1. SHP2 is also known to dephosphorylate STAT1 at both the tyrosine and serine residues in nuclei [13], therefore, quercetin may inhibit SHP2 to cause direct activation of STAT1. Remarkably, it is relatively easy to increase the plasma concentration of quercetin in human to above 10 μM, through supplementation with quercetin or quercetin-enriched foods without any significant toxicity effects [30]. Clinically, type I IFNs have been used in the adjuvant setting after resection of human hepatocellular carcinoma [31]. Moreover, SHP2 overexpression is found to enhance liver cancer progression and predict poor prognosis of patients [32]. Based on our results, the quercetin inhibition of SHP2, together with the potentiation of the IFNα-induced JAK/STAT pathway signaling at low concentrations (1-10 μM), heralds a novel mechanism to explain the anticancer effects of quercetin at a physiological level.

Quercetin is known to inhibit several biomarkers involved in the development of inflammation and cancers [18, 30]. Although quercetin inhibits ERK, a key kinase in the development of inflammation and tumor, however, the mechanism responsible for this effect has not been properly elucidated [33, 34]. The inactivation of protein phosphatase SHP2 has been known to inhibit the activation of RAS-ERK [9]. In the present study, quercetin was observed to inhibit SHP2, thus explaining its inhibitory effects on ERK activation and pro-inflammatory cytokine production. Moreover, quercetin was found to inhibit the cell growth of several human cancer types by reducing the activity and expression of proteins from the EGFR family [35]. Some studies have demonstrated the obligatory requirement of SHP2 in multiple oncogenic receptor tyrosine kinase pathways, including EGFR [36]. Cancer cell lines dependent on known receptor tyrosine kinases (such as EGFR, ERBB2, c-MET, and FLT3) were found to be sensitive to SHP2 depletion [37]. Therefore, inhibition of the SHP2 by quercetin may disturb EGFR and related oncogenic signals and increase its antitumor activity, as observed for other inhibitors of SHP2 [38]. Quercetin was shown to induce cancer cell apoptosis by increasing the stability of p53, a pivotal tumor suppressor associated with inhibition of cell proliferation; again, the mechanism of action was unclear [39–42]. Through a signaling cascade including p53, the inhibition of SHP-2 can induce the senescence and inhibition of the self-renewal of tumor cells and blockage of tumor formation and growth. Hence, the pro-apoptotic effect of quercetin on cancer cells may be attributed to its inhibition on the activity and expression of SHP2 in a p53-dependent manner.

Epidemiological studies indicate that long-term consumption of foods and vegetables rich in flavonoids reduces the risk of chronic diseases, especially cancer [43]. Quercetin, one of the most abundant dietary flavonoids, has been shown to exert several anticancer and anti-inflammatory effects. In spite of various studies that suggest the cytotoxicity of quercetin in various cancer cell lines, the mechanism of action is largely unclear [18]. In a previous report, we identified luteolin, emodin, apigenin, and quercetin as JAK/STAT pathway activators [5]. Luteolin was found to activate JAK/STAT pathway via PKA-mediated inhibition of SHP2 [6]. Emodin and apigenin were found to activate the JAK/STAT signaling by 26S proteasome inhibition and IFNAR1 stabilization [44, 45]. In this study, quercetin was further found to enhance INF-α-induced activation of JAK/STAT signaling by direct inhibition of SHP2, which suggested that flavonoids may activate the JAK/STAT pathway through a series of distinct mechanisms. Patients receiving immunotherapies are recommended to maintain a healthy diet with a sufficient intake of vitamins and minerals to boost the functioning of the immune system, despite the potential mechanisms of this enhancement remaining largely unknown [46]. SHP2 is a key mediator of the PD-1 and BTLA immune checkpoint pathways [10]. The inactivation of the JAK/STAT pathway was associated with the acquired resistance of PD-1 blockage therapy in melanoma [47]. Currently, IFN-α/β are used in combination with conventional chemotherapeutics, targeted anticancer agents, and immunostimulatory agents such as checkpoint blockers in clinical trials [48]. Therefore, the inhibition of SHP-2 by quercetin or quercetin-rich products as a dietary supplement may be beneficial to enhance the IFN-induced anticancer effects during immunotherapy, which is a valuable topic for further study.

In conclusion, quercetin, a major constituent in dietary flavonoids, promoted the inhibitory activity of IFN-α on tumor cell proliferation through the JAK/STAT pathway activation by inhibition of SHP2. This study proposed a novel mechanism to explain the anticancer effects of quercetin, which necessitates further exploration as a promising adjuvant for type I IFN therapy. The consumption of quercetin or quercetin-rich products as a dietary supplement may boost the anticancer effect of type I IFNs or immune checkpoint blockers during cancer immunotherapy, which presents a worthwhile topic for further study.

## MATERIALS AND METHODS

### Reagents and plasmids

Quercetin was obtained from Solarbio Science & Technology Co. Ltd (Beijing, China), the SHP2 inhibitor (NSC-87877) was obtained from Tocris Bioscience (MN, USA), and fluorogenic 6, 8-difluoro-4-methylumbelliferyl phosphate (DiFMUP) was purchased from Invitrogen (Carlsbad, CA, USA). The compounds were dissolved in dimethyl sulfoxide (DMSO; Sigma) and stored in small volume aliquots at −20 °C. IFN-α (recombinant human IFN-α2a) was purchased from ProSpec-Tany Techno Gene Ltd (Shanghai, China). The lyophilized protein was reconstituted in sterile water with carrier protein (0.1% bovine serum albumin) at a concentration of 100 μg/mL and stored at −80 °C. The pCMV-SHP-2 plasmid was from Fisher Scientific (Pittsburgh, PA, USA) and we obtained Trans-EZ transfection reagent from Sunbio Medical Biotechnology (Shanghai, China).

### Cell culture and transfection

Human hepatocellular carcinoma cell lines (HepG2 and Huh-7) and human embryonic kidney (HEK293A) cells were purchased from Cell Bank of Chinese Academy of Science (Shanghai, China). HepG2 cells were maintained in Roswell Park Memorial Institute (RMPI; Hyclone, UT, USA) supplemented with 10% fetal calf serum (Invitrogen) and 1% penicillin/streptomycin. Huh-7 and HEK293A cells were maintained in Dulbecco’s modified Eagle’s medium (DMEM; Hyclone, UT, USA) at 37 °C in an atmosphere of 5% CO_2_. The HepG2-ISRE-Luc2 cell line was established and maintained as previously reported [5]. For all SHP2 overexpression experiments, transfection was performed 18 h following seeding. Two hours before transfection, confluent cells were cultured in fresh medium at 37 °C for 2 hours. plasmids (2 μg/mL) were transfected into cells by using Trans-EZ transfection reagent. After 6 h, the transfection complex was replaced with fresh medium. The transfected cells were then used for subsequent studies after 24 h.

### Luciferase reporter assay

HepG2-ISRE-Luc2 cells were seeded at 1 × 10^4^ cells/well in 96-well plates and incubated for 24 h. The cells were pretreated with various concentrations of quercetin for 2 h and then 200 U/mL IFN-α was added for a further 24 h. The luciferase activity of the total cell lysate was measured using a Luciferase Reporter Assay System (Promega) in accordance with the manufacturer’s instructions. The luminescence intensity was measured by using a Thermo Scientific Varioskan^®^ Flash (Thermo Scientific, USA).

### Western blot analysis

After treatment, the cells were harvested in RIPA buffer supplemented with a protease inhibitor cocktail (Sigma, Shanghai, China). The protein concentration was measured by using a BCA protein assay kit (Bestbio, Shanghai, China). Aliquots of total cell lysates (50 μg protein) were mixed with loading buffer, boiled for 5 min, and separated on a 10% sodium dodecyl sulfate (SDS)-polyacrylamide gel. After electrophoresis, the proteins were transferred to nitrocellulose membranes. The membranes were blocked with 5% bovine serum albumin or 5% de-fatted milk powder for 1 h at room temperature and then incubated overnight at 4 °C with each of the following antibodies: anti-phospho-STAT1 (SAB Signalway Antibody, College Park, MD, USA), anti-STAT1 (Proteintech, IL, USA), anti-phospho-STAT2 (SAB), anti-STAT2 (Proteintech), anti-phospho-STAT3 (SAB), anti-STAT3 (Proteintech), anti-SOCS1 (Proteintech), anti-SOCS3 (Proteintech), anti-SHP1 (Proteintech), anti-SHP2 (Proteintech), anti-GAPDH (Huabio, Hangzhou, China), anti-Jak1 (SAB), anti-phospho-Jak1 (SAB), anti-Tyk2 (Proteintech), and anti-phospho-Tyk2 (SAB). Thereafter, the membranes were incubated with an appropriate peroxidase-conjugated secondary antibody. The presence of the secondary antibody was determined by the detection of enhanced chemiluminescence solution (Amersham Biosciences, Piscataway, NJ, USA).

### Immunoprecipitation

HEK293A cells were lysed in RIPA buffer that contained a complete protease inhibitor mixture and phosphatase after treatment with quercetin and SHP2 inhibitor (NSC-87877) for 6 h. The lysates were incubated on ice for 10 min and centrifuged at 20,000 × *g* for 15 min at 4 °C. Cleared lysates were incubated with SHP2 antibody overnight at 4 °C with agitation, followed by precipitation of antibody-antigen complexes with protein A/G-agarose (Santa Cruz Biotech, TX, USA). The immunoprecipitates were washed three times and then the complexes were resuspended gently in reaction buffer (100 μL) and transferred to a 96-well plate.

### Recombinant SHP2 expression and activity assay

The plasmid for the expression of glutathione S-transferase (GST)-SHP2 (residues 205–593) fusion protein was constructed in pGEX-4T-1 (Madison WI, USA) by using PCR subcloning techniques. The recombinant plasmid was verified by DNA sequencing. The vector was transformed into *Escherichia coli* BL21 and grown overnight in LB medium (300 mL) containing ampicillin (100 mg/L). Once the culture reached an OD600 of 0.6, protein expression was induced by the addition of 1 mM isopropyl β-D-1-thiogalactopyranoside (IPTG) to the culture, which was then maintained for 4 h at 37 °C. Cells were pelleted by centrifugation for 20 min at 10,000 × g and resuspended in PBS (20 mL) containing lysozyme and phenylmethanesulfonyl fluoride. The clarified lysate was then filtered through a 45-μm syringe filter and affinity purified with glutathione-Sepharose. GST-SHP2 was eluted from the column with 3 mL elution buffer (50 mM Tris-HCl, pH 8.0, 10 mM glutathione, 2 mM EDTA, and 2 mM DTT) and 500 μL fractions were collected. The fractions containing the highest SHP-2 activity were pooled and dialyzed against dialysis buffer (10 mM Tris-HCl, pH 7.5, 1 mM EDTA, 2 mM DTT, 30% glycerol) overnight using a 3500 MWCO Slide-A-Lyzer Dialysis Cassette (Thermo Fisher Scientific, USA). Recombinant SHP1 was purchased from Sino Biological (Beijing, China).

SHP2 and SHP1 activities were measured using the fluorogenic DiFMUP as the substrate. Each reaction included 5 μL of the test compound or DMSO (solvent) and the reaction buffer, which contained 25 mM MOPS, pH 7.0, 50 mM NaCl, 0.05% Tween 20, 1 mM DTT, 20 μM DiFMUP, 10 nM Microcystin LR, and 20 nM SHP2 protein, in a total volume of 100 μL, in black 96-well plates. The reaction was initiated by the addition of DiFMUP and incubated for 30 min at 37°C. The DiFMUP fluorescence signal was measured at an excitation wavelength of 355 nm and an emission wavelength of 460 nm with a plate reader (Thermo Scientific Varioskan^®^ Flash). The IC_50_ was defined as the concentration of an inhibitor that caused a 50% decrease in enzyme activity. Determination of the IC_50_ was conducted following the measurement of six concentrations of quercetin. The ranges of quercetin concentrations used in each activity assay were determined from preliminary trials. Each experiment was performed in triplicate, and the IC_50_ data were derived from at least three independent experiments. The curve-fitting program Prism 4 (GraphPad Software, San Diego, CA, USA) was used to calculate the IC_50_ values.

### Cellular thermal shift assay

HEK293A cells were harvested and diluted in PBS supplemented with complete protease inhibitor cocktail. The cell suspensions were freeze-thawed three times with liquid nitrogen and the soluble fraction (the lysate) was separated from the cell debris by centrifugation at 20,000 × *g* for 20 min at 4 °C. The cell lysates were diluted with PBS and divided into two aliquots; one aliquot was treated with DMSO and the other aliquot was treated with quercetin (100 μM). Both aliquots were incubated for 30 min at room temperature, and then subsequently divided into 50-μL aliquots and heated individually at different temperatures for 3 min (Eppendorf Mastercycler, Hamburg) followed by a cooling period of 3 min. The appropriate temperatures were determined in preliminary experiments (data not shown). The heated lysates were centrifuged at 20,000 × *g* for 20 min at 4 °C in order to separate the soluble fractions from the precipitates. The supernatants were dissolved in loading buffer, boiled for 5 min, separated by sodium dodecyl sulfate polyacrylamide gel electrophoresis (SDS-PAGE) and analyzed by western blotting. The dose effect of quercetin on the stability of SHP2 and vinculin were evaluated in the same manner.

### Computational binding simulation

Molecular docking was simulated as described below. Briefly, the 3D crystal structure of SHP2 was downloaded from Protein Data Bank (www.rcsb.org) with the PDB code 3B7O (PTPN11, SHP2 D1). This crystal structure has an accessible active site without the N-SH2 and C-SH2 domains. The crystal structure information of quercetin was obtained from a previous report [49]. The structures of SHP2 and quercetin were then prepared using AutoDockTools by removing the bond disorder, adding hydrogen, and deleting water. Docking simulations were carried out with Autodock 4.2.6 and AutodockVina 1.1.2. Docking was performed sequentially in two steps. Initially, a large grid map to cover the whole protein was used to perform blind docking with a grid-point spacing of 0.2 Å. Then, the second step involved localized docking with a smaller grid box (20 Å × 20 Å × 20 Å) centered at the potential binding site of interest, which included the active site and the allosteric site. For Autodock, the docking calculation was performed using the Lamarckian genetic algorithm with default parameters. Cluster analysis was conducted to assess the optimal binding pose of the ligands. For AutodockVina, the default parameters were also used. The docking results were predicted based on the binding energy and the inhibition constant. Additionally, a schematic plan of ligand interactions showing hydrogen bonds and hydrophobic interactions were visualized by LigPlot+. Finally, images were rendered with PyMOL 1.5.0.4 and the best pose was used to calculate the hydrogen bond distances, which were measured between the hydrogen and its assumed binding partner.

### Quantitative real-time reverse transcription-polymerase chain reaction (qRT-PCR)

Total cellular RNA was isolated from treated HepG2 cells using TRIzol reagent (Invitrogen) in accordance with the manufacturer’s protocol. Total RNA was reverse-transcribed using SuperScript III Reverse Transcriptase (Invitrogen) with oligo dT18 primer. Equal amounts of cDNA were subjected to real-time quantitative PCR with the fluorescent dye SYBR Green I in accordance with the manufacturer’s protocol (TransGen Biotech, Beijing, China). The primer pairs used in the assay for interferon-induced double-stranded RNA-activated protein kinase (PKR), 2′,5′-oligoadenylate synthetase (2′,5-OAS), and glyceraldehyde-3-phosphate dehydrogenase (GAPDH) were as follows: PKR, 5′-GTT TGC TTC TCT GGC GGT CTT-3′ (forward), 5′-GCC ATT TCT TCT TCC CGT ATC C-3′ (reverse); 2′,5′-OAS, 5′-AGG TGG TAA AGG GTG GCT CC-3′ (forward), 5′-ACA ACC AGG TCA GCG TCA GAT-3′ (reverse); and GAPDH, 5′-TGC ACC ACC AAC TGC TTA GC-3′ (forward), 5′-GGC ATG GAC TGT GGT CAT GAG-3′ (reverse). The samples were run in triplicate and the relative expression levels of PKR and 2′,5′-OAS were determined by normalizing the expression of each target gene to that of GAPDH using the 2^-ΔΔCt^ method.

### Cell viability assay

HepG2 and Huh-7 cells were plated at 5 × 10^3^ cells/well in 96-well plates in 100 μL medium. The cultured cells were then treated with various concentrations of quercetin or a combination of quercetin and IFN-α. After 72 h, 10 μL of Alamar Blue reagent (SunBio Medical Biotechnology, Shanghai, China) was added to the medium and incubated for 2–4 h until the blue color changed to pink. The relative fluorescence intensity in each well was measured using a Varioskan^®^ Flash.

### Colony formation assay

HepG2 cells (200 cells/well) were seeded in six-well plates, treated with quercetin in combination with IFN-α, and allowed to grow for 10 days to form colonies. The cell colonies were then fixed with methanol at 4 °C for 20 min and stained with crystal violet (0.1% in H_2_O) for 5 min. The plates were rinsed with water, air-dried, photographed, and evaluated for colony formation. Colonies containing more than 50 cells were counted.

### Statistical analysis

Statistical analyses were performed with GraphPad Prism 5.0 software (GraphPad, La Jolla, CA, USA). All experiments were repeated at least three times and representative results were presented. The data were compared by one-way ANOVA followed by Dunnett’s post-hoc test. Differences were considered statistically significant for values of p < 0.05.

## SUPPLEMENTARY MATERIALS FIGURES



## Abbreviations

2′5′-OAS: 2’-5’-oligoadenylate synthetase
DiFMUP: 6, 8-difluoro-4-methylumbelliferyl phosphate
EGFR: epidermal growth factor receptor
IFN: interferon
IFNAR: interferon-α/β receptor
ISRE: interferon-sensitive response element
JAK: janus kinase
PIAS: protein inhibitors of activated STAT
PKA: protein kinase A
PKR: protein kinase R
SHP2: Src homology domain 2 containing tyrosine phosphatase-2
SOCS: suppressor of cytokine signaling
STAT: signal transducer and activator of transcription
