# Supplementary material for: Dietary quercetin potentiates the antiproliferative effect of interferon-α in hepatocellular carcinoma cells through activation of JAK/STAT pathway signaling by inhibition of SHP2 phosphatase
Source: Oncotarget. 2017 Nov 20;8(69):113734–48. doi: 10.18632/oncotarget.22556 (PMC5768359; doi:10.18632/oncotarget.22556)
Supplement: Supplementary file 1 [file oncotarget-08-113734-s001.pdf]

Dietary quercetin potentiates the antiproliferative effect of interferon-α in hepatocellular carcinoma cells through activation of JAK/STAT pathway signaling by inhibition of SHP2 phosphatase

SUPPLEMENTARY MATERIALS

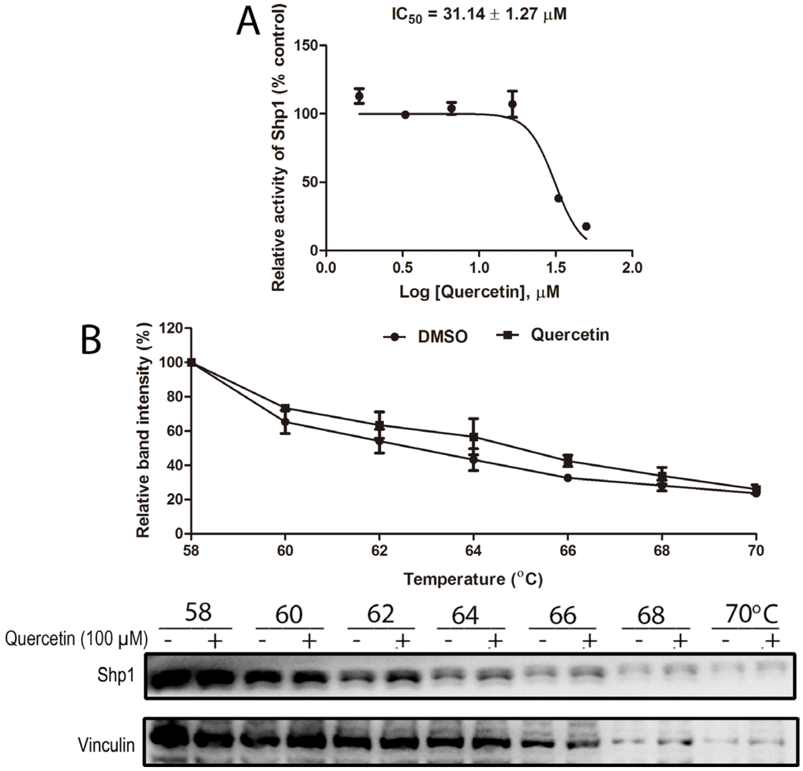

Supplementary Figure 1: Effect of quercetin on SHP1 activity. **(A)** DiFMUP substrate was treated with various concentrations of quercetin in the presence of recombinantly expressed SHP1 protein and the IC_{50} was calculated. **(B)** The cellular thermal shift assay was performed on HEK293A cells as described in Materials and Methods section. The stabilization effect of quercetin on SHP2 and vinculin at different temperatures.

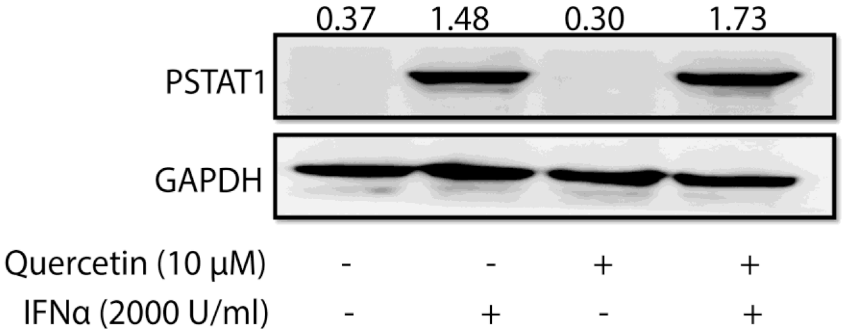

Supplementary Figure 2: Effect of quercetin on STAT1 activation. HepG2 cells were treated with or without quercetin (10 µM) for 6 h, followed by the addition of 2000 U/mL IFN-α for 30 min. The cell lysates were immunoblotted with phospho-STAT1 (Tyr701). GAPDH staining is shown as a loading control.

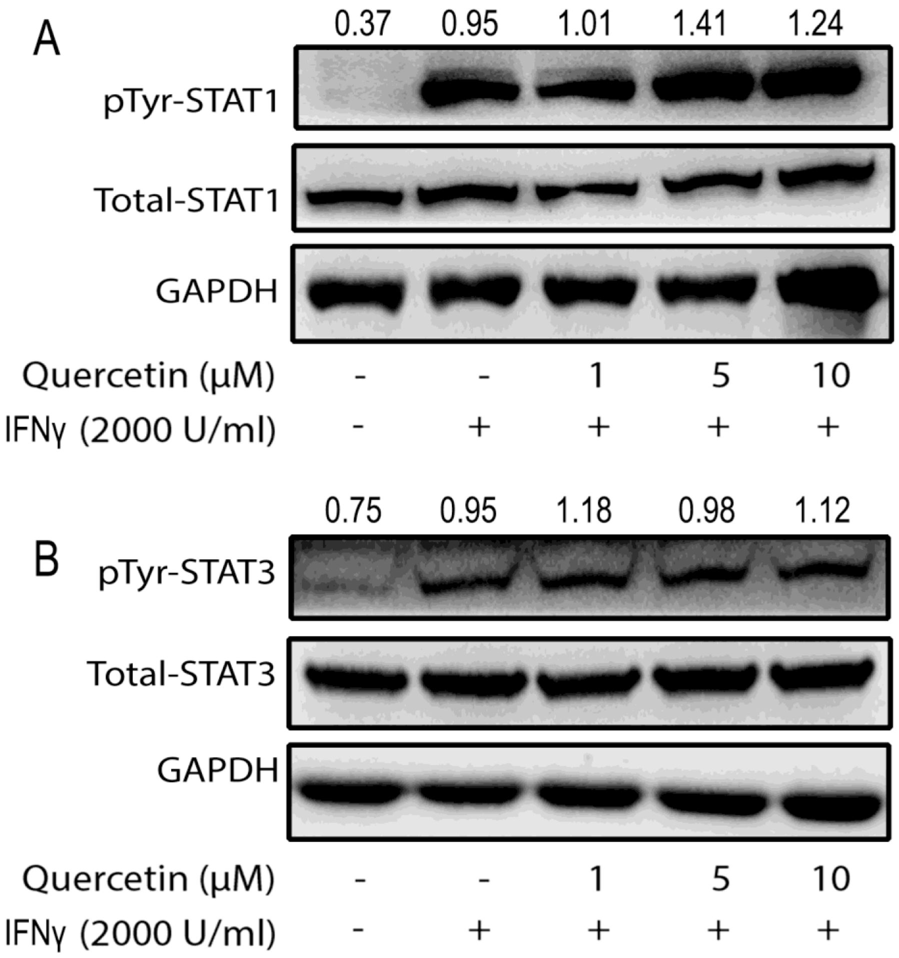

Supplementary Figure 3: Effect of quercetin on IFN-γ-induced STAT1/STAT3 activation. HepG2 cells were treated with or without quercetin (1, 5, and 10 μM) for 6 h, followed by the addition of 2000 U/mL IFN-γ for 30 min. The cell lysates were immunoblotted with phospho-STAT1 (Tyr701, **A**) and STAT3 (Tyr705, **B**). GAPDH staining is shown as a loading control.

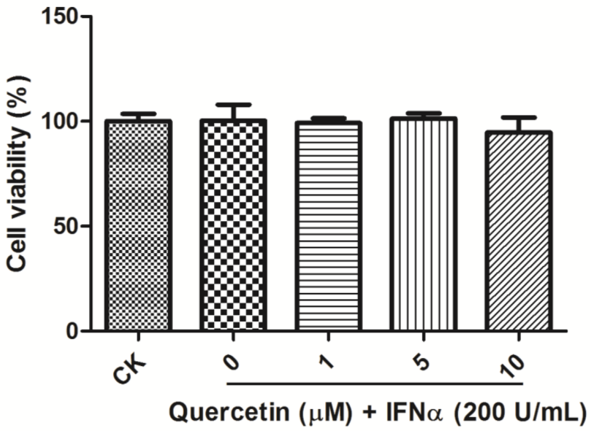

Supplementary Figure 4: Effect of quercetin and IFN-α on cell viability of HepG2-ISRE-Luc2 cells. HepG2-ISRE-Luc2 cells were seeded in 96-well plates (1×10[4] cells/well) and pretreated with various concentrations of quercetin for 2 h before 200 U/mL IFN-α was added for a further 24 h. The cell viability of the total cell was measured by Alamar Blue.

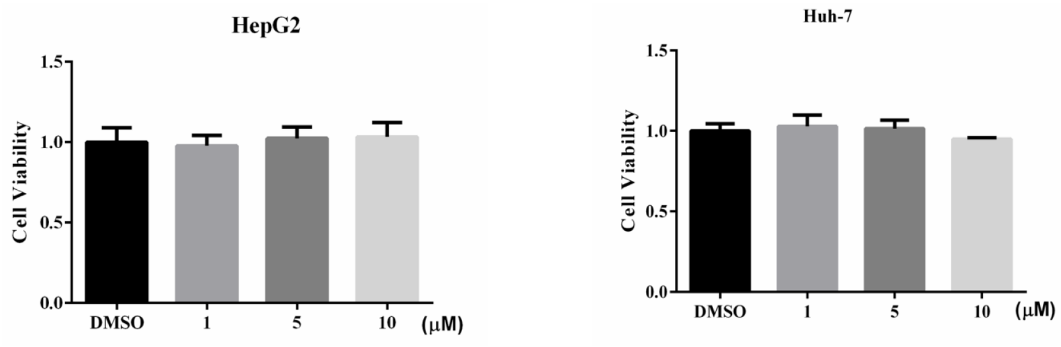

Supplementary Figure 5: Effect of quercetin on cancer cell viability. HepG2 and Huh-7 cells were seeded in 96-well plates at 0.5×10^4 cells/well and treated with various concentrations of quercetin for 72 h. Cell viability was measured using the Alamar Blue assay and the values are expressed as the percentage cell viability relative to the DMSO control.

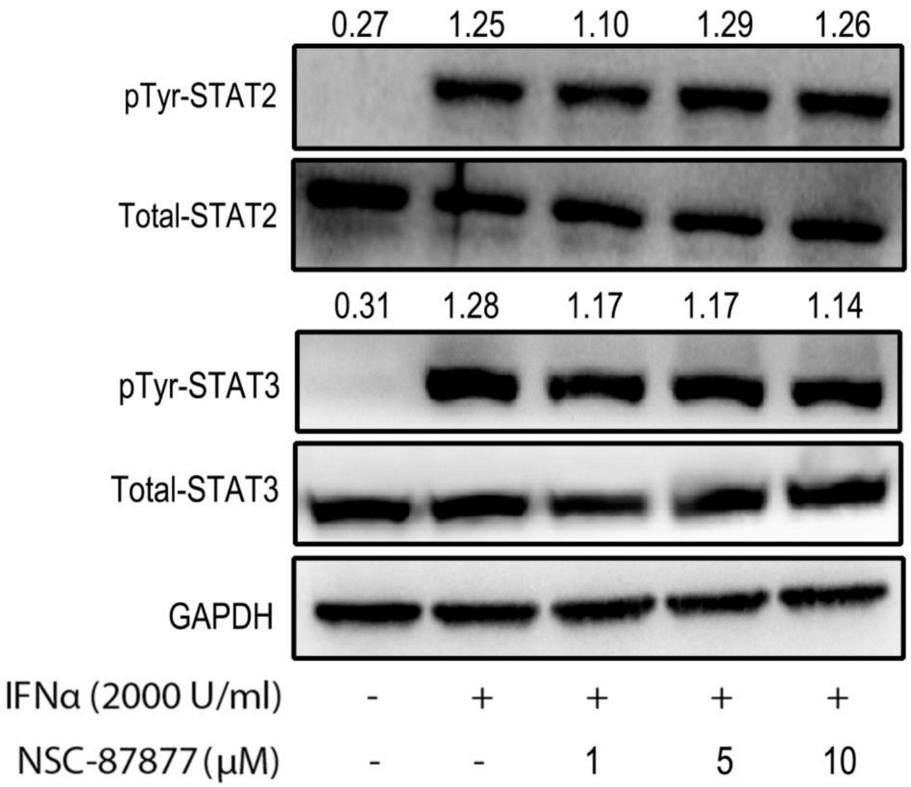

Supplementary Figure 6: Effect of NSC-87877 on IFN-α-induced STAT2/STAT3 activation. HepG2 cells were treated with or without NSC-87877 (1, 5, and 10 μM) for 6 h, followed by the addition of 2000 U/mL IFN-α for 30 min. The cell lysates were immunoblotted with phospho-STAT2 (Tyr690) and phospho-STAT3 (Tyr705). GAPDH staining is shown as a loading control.

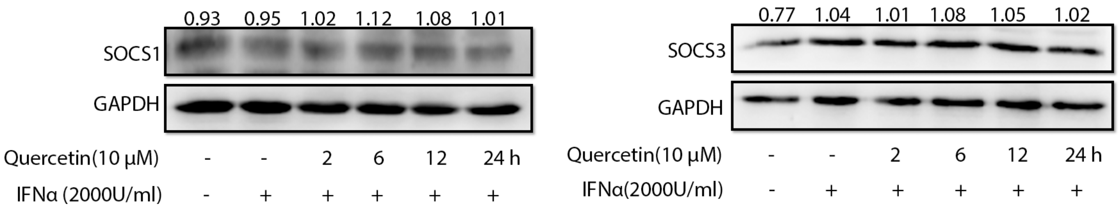

Supplementary Figure 7: Effect of quercetin on SOCS1/3 expression. HepG2 cells were treated with quercetin (10μM) for 2, 6, 12 and 24 h, followed by the addition of 2000 U/mL IFN-α for 30 min. The cell lysates were immunoblotted with SOCS 1 and SOCS 3 antibody. GAPDH staining is shown as a loading control.

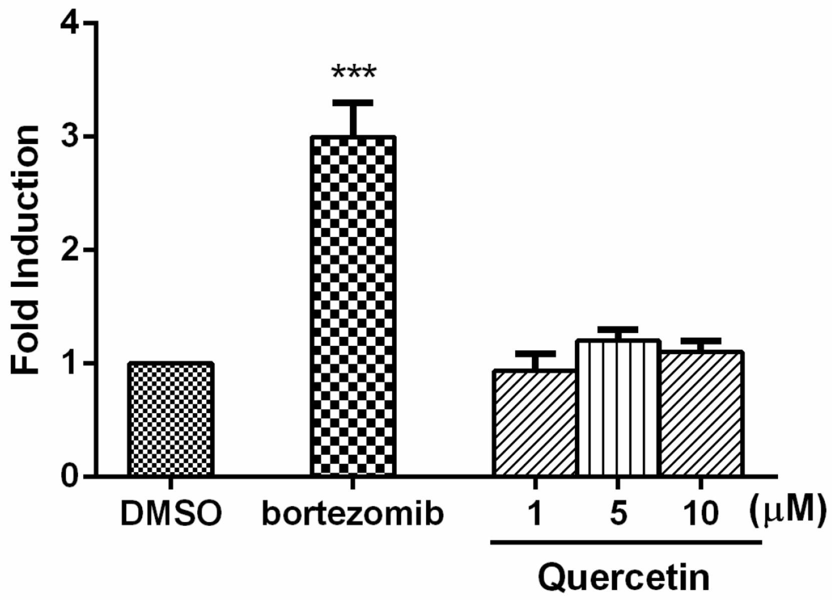

Supplementary Figure 8: Effect of quercetin on 26S proteasome. HEK293A-luciferase-cODC cells [48] were seeded in 96-well plates and treated in the presence of the indicated concentrations of emodin and 1 μM bortezomib for 3 h.
